# Rapid identification of apolipoprotein E genotypes by high-resolution melting analysis in Chinese Han and African Fang populations

**DOI:** 10.3892/etm.2014.2097

**Published:** 2014-12-01

**Authors:** XIU-HUI ZHAN, GUANG-CAI ZHA, JI-WEI JIAO, LI-YE YANG, XIAO-FEN ZHAN, JIANG-TAO CHEN, DONG-DE XIE, URBANO MONSUY EYI, ROCIO APICANTE MATESA, MAXIMO MIKO ONDO OBONO, CARLOS SALA EHAPO, ER-JIA WEI, YU-ZHONG ZHENG, HUI YANG, MIN LIN

**Affiliations:** 1 Department of Biology, Hanshan Normal University, Chaozhou, Guangdong 521000, P.R. China; 2 Laboratory Medical Center, Chaozhou Central Hospital Affiliated to Southern Medical University, Chaozhou, Guangdong 521000, P.R. China; 3 The Chinese Medical Aid Team to Equatorial Guinea, Guangzhou, Guangdong 510000, P.R. China; 4 Central Blood Transfusion Service, Malabo Regional Hospital, Malabo 999115, Equatorial Guinea; 5 Department of Internal Medicine, First Affiliated Hospital of Shantou University Medical College, Shantou, Guangdong 515041, P.R. China

**Keywords:** apolipoprotein E, genotype, high-resolution melting, Chinese Han, African Fang

## Abstract

Apolipoprotein E (APOE) gene polymorphism can affect APOE gene transcription, serum lipid levels and repair of tissue damage, which could place individuals at serious risk of cardiovascular disease or certain infectious diseases. Recently, high-resolution melting (HRM) analysis was reported to be a simple, inexpensive, accurate and sensitive method for the genotyping or/and scanning of rare mutations. For this reason, an HRM analysis was used in the present study for APOE genotyping in the Southern Chinese Han and African Fang populations. A total of 100 healthy Southern Chinese Han and 175 healthy African Fang individuals attended the study. Polymerase chain reaction-DNA sequencing was used as a reference method for the genotyping of these samples. The six APOE genotypes could all be rapidly and efficiently identified by HRM analysis, and 100% concordance was found between the HRM analysis and the reference method. The allele frequencies of APOE in the Southern Chinese Han population were 7.0, 87.5 and 5.5% for ɛ2, ɛ3 and ɛ4, respectively. In the African Fang population, the allele frequencies of APOE were 24.3, 65.7 and 10.0% for ɛ2, ɛ3 and ɛ4, respectively. A statistically significant difference was found between the allele frequencies between the populations (P<0.05). In conclusion, the present study revealed the molecular characterization of APOE gene polymorphism in the Han population from the Chaozhou region of Southern China and the Fang population from Equatorial Guinea. The findings of the study indicated that HRM analysis could be used as an accurate and sensitive method for the rapid screening and identification of APOE genotypes in prospective clinical and population genetic analyses.

## Introduction

Apolipoprotein E (APOE) is an important plasma protein involved in lipoprotein metabolism and the transport of cholesterol and triglyceride (1–3). There are three types of common variant alleles (ɛ2, ɛ3 and ɛ4) in the world, which result from two single nucleotide polymorphisms (rs429358 and rs7412) on the APOE gene. These variant alleles can affect APOE gene transcription and serum levels of cholesterol and triglyceride (4). Epidemiological studies have indicated that there is a notable association between APOE gene polymorphism and a serious risk of cardiovascular disease or certain infectious diseases (4–6). Individuals inherit one allele of APOE from each of their parents, thus yielding six possible genotypes: ɛ2/ɛ2, ɛ2/ɛ3, ɛ2/ɛ4, ɛ3/ɛ3, ɛ3/ɛ4 and ɛ4/ɛ4 (7). The frequency of APOE genotypes varies among ethnic groups, but wild-type ɛ3/ɛ3 is the most frequent genotype in all populations (8,9).

Various methods have been developed to detect APOE genotypes, including allele-specific polymerase chain reaction-restriction fragment length polymorphism (PCR-RFLP) analysis (10,11), PCR-single-strand conformational polymorphism analysis (12), microarrays (13), PCR-DNA sequencing (14) and allele-specific PCR (15). These approaches, however, are expensive or time-consuming and are thus not appropriate for rapid molecular diagnoses in clinical practice or for molecular screening in large populations; therefore, the development of a reliable and rapid method of detecting the common APOE genotypes would be useful for clinical and population genetic analyses. High-resolution melting (HRM) analysis is a novel, rapid and powerful mutation screening technique in which PCR and mutation scanning are performed simultaneously in a single procedure lasting <30 min. In the present study, an HRM assay was developed to identify APOE genotypes rapidly and effectively in the Chinese Han and African Fang populations.

## Materials and methods

### Population samples

The study subjects were collected from two ethnic groups: Between February and December 2012, 100 unrelated healthy Southern Han Chinese individuals (50 male and 50 female) attended the study in the Chaozhou region of China (Guangdong, China), and between February and October 2012, 175 unrelated healthy African Fang individuals (87 male and 88 female) attended the study on Bioko Island (Equatorial Guinea). Ethical approval to undertake the survey was obtained from the Ethics Committees of the Malabo Regional Hospital (Malabo, Equatorial Guinea) and the Chaozhou Central Hospital Affiliated to Southern Medical University (Chaozhou, China). The ages of the subjects ranged from 20 to 65 years. Information sheets with nationality, gender, age and aboriginal status and written consent forms were available in Chinese or Spanish to ensure comprehensive understanding of the study objectives, and informed consent was signed or thumb-printed by the participants. Subsequent to obtaining informed consent, 2-ml peripheral blood samples were collected into tubes with EDTA-K_2_ by the medical laboratories in the Chaozhou Central Hospital or Malabo Regional Hospital for storage at 4°C until required.

### Strategy for study

**Figure 1:**
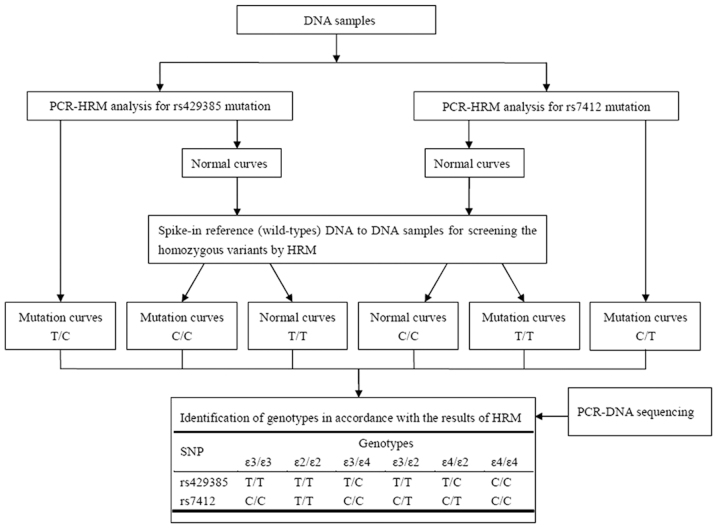
Strategy for the identification of apolipoprotein E genotypes in the study. PCR, polymerase chain reaction; HRM, high-resolution melting; SNP, single nucleotide polymorphism.

A strategy was adopted for detecting the APOE gene polymorphism (Fig. 1). Firstly, the heterozygote and homozygote were identified with each of two paired primers (Table I) by HRM assay. Secondly, since the melting curve shapes of the homozygous variants were similar to those of the wild-type, homozygous DNA samples were mixed with the same amount of reference DNA (wild-types ɛ3/ɛ3) to generate the heteroduplex, thus making it easy to separate the homozygous mutations from the wild-types. The results of the HRM were then analyzed for the identification of the APOE genotypes. Finally, all amplicons were again ascertained by DNA sequencing.

### DNA isolation

Genomic DNA was extracted from peripheral blood leukocytes by the DNA blood mini kit (Qiagen Co. Ltd., Shanghai, China). The DNA concentration was determined using an ultraviolet spectrophotometer [Unico (Shanghai) Instruments Co., Ltd., Shanghai, China] at a wavelength of 260 nm. All DNA templates were adjusted to 50 ng/μl concentration. The DNA samples were stored at −80°C until required and would be used for the subsequent HRM analysis and DNA sequencing.

### APOE genotyping by HRM analysis

Oligo 6.64 (Molecular Biology Insights Inc., Cascade, CO, USA) and Primer Premier 5.0 (Premier Biosoft, Palo Alto, CA, USA) software were used for primer design. Two sets of PCR primers were designed to amplify the regions encompassing rs7412 [Human genome variation society (HGVS) name: NC_000019.9:g.45412079C>T] and rs429358 (HGVS name: NC_000019.9:g.45411941T>C). The amplification length and localization of all primers are indicated in Table I. The synthesized primers were all of standard molecular biology quality (Shanghai Invitrogen Biotechnology Co. Ltd, Shanghai, China).

PCR amplification was carried out with LightCycler 480 II (Roche Diagnostics GmbH, Mannheim, Germany). For the PCR reaction, each tube contained, in a final volume of 20 μl, 100 ng genomic DNA, 100 μM each deoxynucleotide triphosphate (dNTP), 0.2 μM each primer, 1.0 μl LC Green Plus^®^ (Idaho Technology Inc., Salt Lake City, UT, USA), 4.0 μl 5X PCR buffer, 0.5 units HotStart Taq DNA polymerase (Takara, Dalian, China) and 9.2 μl double-distilled H_2_O. The reaction conditions were 95°C for 5 min, followed by 35 cycles at 98°C for 10 sec and 68°C for 20 sec.

Following amplification, the samples were incubated at 95°C for 1 min and then at 40°C for 1 min. Melting curve profiles were generated by increasing the temperature from 65 to 95°C, and fluorescence was continuously acquired at a ramping rate of 0.05°C/sec with 25 acquisitions per degree. HRM analysis was performed by the LightCycler 480 SW 1.5 software (Roche Diagnostics GmbH). The samples with known mutations, which had been validated by DNA sequencing, were used as standard references. The plots of samples were identified as the same mutation of the standard when they were classified into the standard reference.

### PCR-DNA sequencing

DNA sequencing of the APOE gene was performed with a set of primers (Table I). The reaction mixture (a volume of 50 μl) consisted of 100 ng genomic DNA, 2.0 mM MgCl_2_, 1.0 μM each primer, 200 μM dNTP, 5 μl 10X PCR buffer and 2.5 units Taq DNA polymerase (Takara). Reactions were carried out in an MJ Mini Personal Thermal Cycler (Bio-Rad Laboratories, Inc., Hercules, CA, USA) with an initial denaturing step of 95°C for 10 min and then 35 cycles of 95°C for 1 min, 58°C for 1 min and 72°C for 1.5 min with a final extension at 72°C for 10 min. A total of 10 μl PCR product was subsequently fractionated on a 1% agar gel to check for the integrity of the products. The PCR products were then sequenced using an ABI 3730xL DNA Sequencer (Perkin-Elmer Applied Biosystems, Norwalk, CT, USA).

### Statistical analysis

Statistical analyses were performed using SPSS (version 16.0) statistical software (SPSS Inc., Chicago, IL, USA). The allele frequencies and genotype distributions were calculated by the gene-counting method (16). The χ^2^ or Fisher’s exact test was used not only to evaluate the allelic and genotypic frequencies, but also to estimate the Hardy-Weinberg equilibrium. P<0.05 was considered to indicate a statistically significant difference.

## Results

### HRM analysis of APOE genotypes

**Figure 2:**
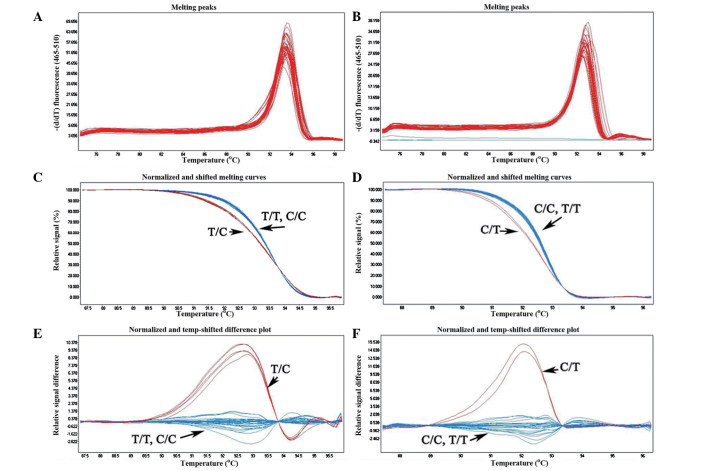
High-resolution melting analysis results of rs429358 and rs7412. (A and B) Tm calling analysis for the amplicon of (A) rs429358 and (B) rs7412. (C and D) Normalized and shifted melting curves for the amplicon of (C) rs429358 and (D) rs7412. (E and F) Normalized and temperature-shifted difference plot for the amplicon of (E) rs429358 and (F) rs7412.

**Figure 3:**
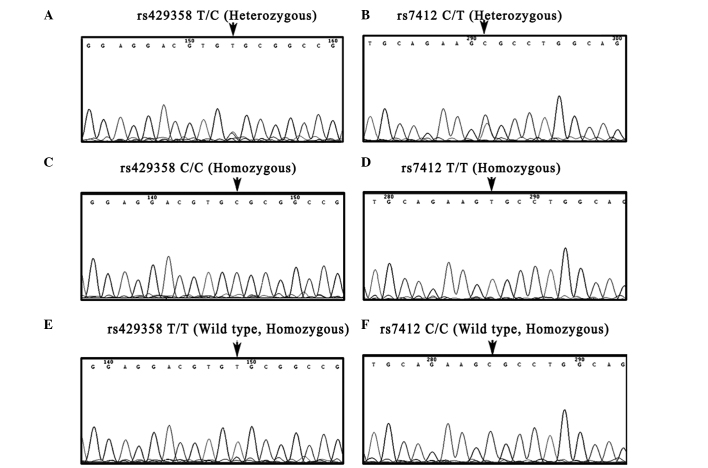
Polymerase chain reaction-DNA sequencing results of rs429358 and rs7412. (A) rs429358 T/C heterozygote; (B) rs7412 T/C heterozygote; (C) rs429358 C/C homozygote; (D) rs7412 T/T homozygote; (E) rs429358 T/T heterozygote (wild-type); (F) rs7412 C/C heterozygote (wild-type).

A total of 275 samples (100 Chinese and 175 African) were analyzed by the HRM method. From Fig. 2A and B, only a single sharp peak was found in the melting curve shapes. This indicated that there was no nonspecific product during the reaction. Heterozygous mutation could be easily distinguished from the wild-type, but the homozygous mutation and wild-type exhibited almost indistinguishable melting curve profiles (Fig. 2C–F); therefore, a strategy was formulated to solve the problem (Fig. 1). Wild-type DNA (ɛ3/ɛ3) was added to produce the heteroduplex DNA, and then the melting curves of the homozygous mutations could be distinguished from those of the wild-types. Compared with the results of the reference method (PCR-DNA sequencing) (Fig. 3), all 275 samples were rapidly and efficiently identified by HRM analysis. The concordance was 100%.

### Frequency distributions of APOE

The frequencies of the APOE genotypes in the Southern Chinese Han and African Fang populations are shown in Table II. The genotype distributions did not deviate from Hardy-Weinberg equilibrium for the population (P>0.05). Consistent with previous reports (5–7), ɛ3/ɛ3 was observed to be the most common genotype in the Southern Han (78%, 78/100) and African Fang (42.9%, 75/175) populations. In addition, no ɛ4/ɛ4 genotype was found in the Southern Chinese Han population.

The allele frequencies of APOE in the Southern Chinese Han population were 7.0, 87.5 and 5.5% for ɛ2, ɛ3 and ɛ4, respectively (Table III). In the African Fang population, the allele frequencies of APOE were 24.3, 65.7 and 10.0% for ɛ2, ɛ3 and ɛ4, respectively (Table III). A statistically significant difference was found between the allele frequencies between the populations (P<0.05).

## Discussion

In previous investigations, PCR-RFLP has been the most common method for APOE polymorphism identification (10,11). The steps of PCR-RFLP include the PCR reaction, treatment of amplified fragments by the restriction enzyme *Hha*I and gel electrophoresis (10,11). As such, this technique is time-consuming and costly for a large-scale analysis. In the present study, an HRM analysis method was adopted for the identification of APOE genotypes. HRM analysis is a more rapid, cost-effective and convenient closed-tube genotyping approach for the screening of genetic disorders (16,17). This technique could not only reduce the contamination risk, but also be applied to a high-throughput gene mutation screening of a large cohort of patients when required (16,17). The present results showed 100% concordance between HRM analysis and the reference method (PCR-DNA sequencing). This indicated that HRM analysis could be used as an accurate and sensitive method for the rapid screening and identification of APOE genotypes.

The APOE allele frequencies in the Chinese Han population, which were collected from the Chaozhou region, were 7.0% for ɛ2, 87.5% for ɛ3 and 5.5% for ɛ4. Compared with other Chinese populations (Table III) (18–22), the APOE gene allele frequencies of the study population were most similar to those of a Taiwanese population (20), but significantly different from those of the Chinese minority ethnic groups: The Uygur population in the Xinjiang Uygur Autonomous Region (22), the Li population on Hainan Island (18) and the Zhuang population in the Guangxi Zhuang Autonomous Region (19). A number of factors may be used to explain this finding. Firstly, the Southern Han population in the Chaozhou region, known as the Fulao peoples, largely comes from Henan and Shanxi via Fujian with the well-maintained language and customs of north-central China. The majority of the Fulao peoples first settled in Fujian, and then migrated to the Chaoshan region. Due to geographic isolation and the historical problems of population migration, the Fulao became a relatively isolated population. Notable genetic similarities have been found between the Chaoshan Han and Fujian Han populations (23). Secondly, Fujian faces Taiwan across the sea. The populations on the two sides of the straits of Taiwan are closely associated since they have the same ancestors, speak the same dialect and share the same customs and cultural traditions. Statistics published in Taiwan (20) have stated that the Taiwanese population is predominantly (80%) comprised of individuals of Fujian origin. We therefore hypothesize that the considerable similarities in APOE allele frequencies are due to the common genetic background shared between the Chaozhou Han and Taiwan Han populations.

The samples from individuals of the African Fang population (an ethnic group of Bantu origin) were collected from Bioko Island in Equatorial Guinea (24,25). The APOE allele frequencies of the Fang population were 24.3% for ɛ2, 65.7% for ɛ3 and 10.0% for ɛ4. The frequency of APOE ɛ2 (24.3%) in the Fang population was higher than almost all the other known values for sub-Saharan African populations (the Pygmy, Nigerian, Sudanese, Ethiopian, Ghanaian and central African populations) (Table III), but the APOE ɛ4 allele frequency (10.0%) was lower than the values for these sub-Saharan African populations (26,27) (Table III). Bioko Island is characterized as a humid tropical environment with hyper-endemic malaria transmission (28). As a significant threat to human life, malaria has exerted the strongest known selection pressure on the human genome in the past 10,000 years since the origination of agriculture. Previous studies have reported that there may be a close association between APOE gene polymorphism and infection with malaria (29–31). For example, a study of the interactions between the proteins of *Plasmodium falciparum* and human APOE indicated a preferential interaction of the *P. falciparum* PFE1590w protein with human APOE ɛ3 and APOE ɛ4, but not APOE ɛ2 (29). This means that individuals carrying APOE ɛ3 and ɛ4 alleles are more likely to develop severe malaria (cerebral malaria and severe anemia) (29); therefore, the higher APOE ɛ2 allele frequency in the Fang population on Bioko Island may be the result of selection due to malaria. This hypothesis requires future studies for its confirmation.

In conclusion, the present study provides the first molecular characterization of the APOE gene polymorphism in the Han population from Southern China and Fang population from Equatorial Guinea. These data could be useful for future genetic investigations of a number of disease risks within the Southern Han and Fang populations. The present results also indicated that HRM analysis could be used as an accurate and sensitive method for the rapid screening and identification of APOE genotypes in prospective clinical and population genetic analyses.

## Figures and Tables

**Table I:** Primers for the HRM assay and polymerase chain reaction-DNA sequencing.

Name	Primers (5′-3′)	Product (bp)
HRM-rs429358-F	CGGGCACGGCTGTCCAAG	91
HRM-rs429358-R	CGCGGTACTGCACCAGGC	
HRM-rs7412-F	GCAAGCTGCGTAAGCGGCTCC	112
HRM-rs7412-R	TCGCGGATGGCGCTGAGG	
Sequencing-F	CCTCCCACTGTGCGACACCCTCC	532
Sequencing-R	GTCCGGCTGCCCATCTCCTCCAT	

HRM, high-resolution melting; F, forward, R, reverse.

**Table II:** Frequencies of apolipoprotein E genotypes in the Southern Chinese Han and African Fang populations.

Genotypes	Southern Chinese Han, n (%)	African Fang, n (%)
ɛ3/ɛ3	78 (78.0)	75 (42.9)
ɛ2/ɛ2	2 (2.0)	1 (0.6)
ɛ3/ɛ4	10 (10.0)	56 (32.0)
ɛ3/ɛ2	9 (9.0)	24 (13.7)
ɛ4/ɛ2	1 (1.0)	9 (5.1)
ɛ4/ɛ4	0 (0.0)	10 (5.7)
Total	100 (100)	175 (100)

**Table III:** Allele frequencies of the apolipoprotein E gene in various populations.

			Apolipoprotein E allele frequencies		
			
First author, year (ref.)	Population	n	ɛ2 (%)	ɛ3 (%)	ɛ4 (%)
Present data	Han (Chaozhou, China)	100	7.0	87.5	5.5
Wang, 2012 (18)	Han (Xinjiang, China)	150	8.1	77.2	14.6
Hu, 2011 (19)	Han (Guangxi, China)	200	9.2	81.4	9.3
Kao, 1995 (20)	Han (Taiwan, China)	564	7.6	87.5	4.9
Wang, 1988 (21)	Han (Beijing, China)	95	5.3	88.3	6.4
Wang, 1988 (21)	Han (Hubei, China)	113	9.3	83.2	7.5
Wang, 1988 (21)	Han (Hunan, China)	102	5.3	88.4	6.3
Wang, 1988 (21)	Han (Jiangsu, China)	168	7.1	86.3	6.6
Mayila, 2005 (22)	Uygur (Xinjiang, China)	163	12.0	82.1	16.7
Hu, 2011 (19)	Zhuang (Guangxi, China)	278	15.2	79.8	4.9
Wang, 2012 (18)	Li (Hainan, China)	50	9.0	76.0	15.0
Present data	African Fang (Equatorial Guinea)	175	24.3	65.7	10.0
Wozniak, 2003 (26)	African (Ghana)	110	14.5	61.4	24.1
Wozniak, 2003 (26)	African (Central African Rep)	70	5.7	53.6	40.7
Wozniak, 2003 (26)	African (1, Nigeria)	97	10.3	74.2	24.1
Wozniak, 2003 (26)	African (2, Nigeria)	781	6.4	68.4	25.2
Wozniak, 2003 (26)	African (Sudan)	103	8.3	62.6	29.1
Wozniak, 2003 (26)	African (Ethiopia)	164	3.0	81.1	15.8
Wozniak, 2003 (26)	African (Morocco)	100	6.5	85.0	8.5
Wozniak, 2003 (26)	African (South Africa)	247	7.7	55.3	37.0
